# Capgras syndrome. Where to find it?

**DOI:** 10.1192/j.eurpsy.2021.1267

**Published:** 2021-08-13

**Authors:** I.D.L.M. Santos Carrasco, J. Gonçalves Cerejeira, E. Rodríguez Vázquez, C. Capella Meseguer, M. Queipo De Llano De La Viuda, G. Guerra Valera, A. Gonzaga Ramírez, C. De Andrés Lobo, C. Vallecillo Adame, T. Jiménez Aparicio, A. Pérez Escudero

**Affiliations:** Psychiatry, Clinical Hospital of Valladolid, Valladolid, Spain

**Keywords:** psychotic depression, Capgras syndrome, delusional misidentification, differential diagnosis

## Abstract

**Introduction:**

Capgras syndrome, where patients have the conviction that one or more close people have been replaced by a “double,” is the most prevalent delusional misidentification syndrome. It appears in psychiatric illness and organic brain damage. It seems to be due to damage of bifrontal and right limbic and temporal regions, mainly in the right hemisphere.

**Objectives:**

To review the pathologies associated to Capgras Syndrome and the relevance of the differential diagnosis

**Methods:**

53-year-old female was admitted due to great sadness, crying, social withdrawal and severe paranoid concerns over the last month. Follow-up in Mental Health since 2014, because of anxious depression. After her mother’s death, she felt being followed because of old faults. Since then, low dosis of antipsychotics were used. Now she is afraid of being harmed in relation to petty thefts she committed over 15 years ago. In recent days, she has been noticing small details indicating that her family members have been impersonated by strangers, showing anguish regarding their whereabouts.

**Results:**

During her admission, high doses of antidepresants and paliperidone 6 mg/day were used with the complete disappearance of Capgras Syndrome and her anguish. Mild guilty thoughts were present after her discharge. That is why she was diagnosed with psychotic depression.

**Conclusions:**

Capgras syndrome can be encountered in primary psychiatric diagnosis (particularly in schizophrenia and mood disorders) – where an organic element may exist in about a third of all cases – or secondary to organic disorders or medication-induced, through to overt organic brain damage, particularly in neurodegenerative disease.

